# The Influence of Doing Shift Work on the Lifestyle Habits of Primary Care Nurses

**DOI:** 10.3390/nursrep12020029

**Published:** 2022-04-12

**Authors:** Iris Forcada-Parrilla, Glòria Reig-Garcia, Laura Serra, Dolors Juvinyà-Canal

**Affiliations:** 1 Primary Health Care Nurse, Institut Català de la Salut, 17003 Girona, Spain; iris.forcada@udg.edu; 2 Department of Nursing, Faculty of Nursing, University of Girona, 17003 Girona, Spain; dolors.juvinya@udg.edu; 3 Health and Health Care Research Group, University of Girona, 17003 Girona, Spain; 4 Research Group on Statistics, Econometrics and Health (GRECS), University of Girona, 17003 Girona, Spain; laura.serra@udg.edu; 5 CIBER of Epidemiology and Public Health (CIBERESP), 17003 Girona, Spain

**Keywords:** nursing, primary health care, shift work, lifestyle behaviors

## Abstract

Nurses’ lifestyle habits play a key role in promoting healthy lifestyles; although, they may not always be entirely healthy and can be influenced by working conditions. This paper aims to analyze the influence of doing shift work on nurses’ lifestyle habits. Participants (*n* = 219) were recruited from 27 primary health care centres in Spain. Data were collected on socio-demographic characteristics, working conditions and lifestyle behaviour, assessed by use of an adhoc questionnaire including validated measures. Descriptive analysis and logistic regression models were performed. A total of 95% of the nurses reported having an adequate diet; 45.2% did not engage in any type of physical activity; and 85.8% did not smoke, especially women. A total of 60.3% did shift work, especially the younger ones (80.8%; *p* < 0.001), these nurses being the ones with the worst food habits (81.8%). In contrast, nurses who did shift work, exercised more days a week (69.5%; *p* < 0.001). The dietary habits of the nurses were adequate. Low tobacco consumption and low adherence to physical activity were observed, especially among women. Shift work harmed eating habits and obesity rates, but was a protective factor in terms of adherence to physical exercise.

## 1. Introduction

Positive lifestyle choices and behaviors are fundamental to achieving good health [1]. Nurses are the most trusted health care professionals in society, and as role model educators, they have enormous potential to play a leading role in the task of improving the health of the population [2]. In this sense, primary care nurses are responsible for managing a large number of health conditions in individuals, providing appropriate health promotion recommendations. 

According to Chung [3], nurses who observe healthy lifestyle practices are more effective in motivating and engaging patients in healthy lifestyles, and this has a positive effect on people’s health. However, previous studies have shown that nurses often lack appropriate health behaviors, such as exercising, eating a healthy diet, and practicing stress reduction activities [4,5]. 

A review of the physical activity levels and diet of U.S. nurses found that the majority reported a poor-quality diet (53–61%) and inadequate physical activity levels (60–74%) [6]. Other studies have described the high prevalence of overweight and obesity among nurses, the poor quality of their diets, and their low levels of physical activity [7,8]. 

Some of the barriers that nurses have identified in relation to these unhealthy habits are: inadequate working conditions, such as shift work or nursing workload; poor eating habits among fellow nurses (peer nurses); and limited availability of healthy food options at work [9,10]. Shift work is a work practice that includes a wide diversity of work schedules with negative consequences for the health of the people who practice it [11]. It alters circadian rhythms, decreases total melatonin secretion, and causes sleep disturbances, physical health problems, and psychological disorders [12,13]. Unfortunately, shift working is a common practice among healthcare professionals, especially among nurses, since caring for people’s health requires round-the-clock supervision [14]. According to Liu [15], nurses who work shifts have a higher prevalence of overweight and obesity, as shift working has negative effects on eating patterns [4] and on adherence to physical activity [16].

Thus, and according to the WHO, workplace characteristics are a key factor with direct implications for the health of professionals [17]. The model proposed by Pender [18] indicates that work environment standards influence health behaviors and, accordingly, Wang [19] asserts that it is the responsibility of managers to create healthy work environments and conditions. 

The work environment of nurses has been the focus of attention of researchers and policy makers around the world, yet most studies have focused on hospital settings. The aim of the present study is to analyze the lifestyle habits of primary care nurses in the health region of Spain and to identify the relationship between these habits and their working conditions, specifically shift work. Knowing this information could help guide potential interventions in primary health care in Spain. 

## 2. Methods

### 2.1. Design

This is a cross-sectional study that has gathered information on nurses’ lifestyle habits in the primary care setting of the Girona health region (Spain) and aims to analyze their relationship with working conditions, specifically shift work. 

### 2.2. Sample

The study population consisted of 322 nurses, representing 100% of the nursing professionals working in some of the 27 participating centers that responded to the questionnaire. The inclusion criteria were: working as a nurse in one of the primary care teams participating in the study, and being in active employment at the time of the study.

Nurses were invited to answer the questionnaire by the director of each primary care centre, who sent them the information about the study and the questionnaire via email. Each primary health care director also had printed questionnaire copies, so nurses could answer them directly on paper or print out the questionnaire attached to the mail. The nurses were given 15 days to answer the questionnaires. After this time, the questionnaires were collected and kept in a sealed envelope by the director of each of the participating centres. 

### 2.3. Instruments

An ad hoc, anonymous and self-administered questionnaire was prepared (Appendix A, Table A1, Table A2 and Table A3), consisting of 2 sections. In the first, sociodemographic and labour variables were collected: age, sex, body mass index, work centre, work category, and years of experience in primary health care and doing shift work. The second part consisted of 8 questions related to the following life habits: smoking, physical exercise, and eating habits. These were measured according to 4 validated questionnaires: the Minnesota Leisure Time Physical Activity Questionnaire in Spanish woman [20]; the Minnesota Leisure Time Physical Activity Questionnaire in Spanish men [21]; Use of a three-day estimate food record, a 72 h recall and a food frequency questionnaire for dietary assessment in a Mediterranean Spanish population [22]; and Validity of two short screeners for diet quality in time-limited settings [23].

### 2.4. Validity and Reliability

Prior to the study, a pilot test was conducted with 25 nurses to evaluate the performance and reliability of the measurement instrument. They all responded to the different questions of the questionnaire without any problems and no changes were required. 

### 2.5. Ethical Considerations

This study was approved by the Primary Health Care Ethics Committee (P13/054). The researchers informed all the participants about the objective of the study. Anonymity was maintained at all times. The completed questionnaires did not contain any personal information that could identify participants. The data were analysed by a researcher and the principles defined in the Declaration of Helsinki were followed. 

The data presented in this study are available on request from the corresponding author. Data are not publicly available due to the privacy term signed by the participants in the informed consent.

### 2.6. Data Analysis

The data obtained from the questionnaires were analyzed using the statistical package IBM SPSS Statistics for Windows version 21.o (IBM CORp. Girona, Spain, Released, 2012). 

The variable of interest in this study was whether nurses did shift work or not, i.e., a binary response variable that only takes two values. 

The explanatory or independent variables used to explain the probability of doing or not doing shift work included categorical variables and continuous variables, and could be divided into three groups. The first group included the worker’s inherent characteristics, such as gender (Male = 1 if male, 2 otherwise) and body mass index. In addition, the worker’s age was also included, classified in 4 groups (≤34 years old, 35–44 years old, 45–54 years old, 55–65 years old). In the second group, we considered environmental variables that might affect workers’ performance, such as geographical factors, e.g., work location (Work Region = 1 if located in the south of the territory; Work Region = 2 if located in the north, and Work Region = 3 if located near the sea). The third group referred to those variables that depended directly on the individual: the number of days per week dedicated to exercise (1 day; 2 days; 3 days or more, or none) and variables related to eating habits (daily or weekly consumption of certain products).

A principal component analysis was performed to work with the variables regarding daily or weekly consumption of certain products. In this way, the 10 initial variables related to weekly food consumption frequencies were reduced to just 3 components in the questionnaire: (a) Saturated fats (butter, fast food, vegetable oils, and pastries); (b) Mediterranean diet (pulses, fish, nuts, and dried fruits), and (c) Animal products (cheese, cold cuts, and meat). The 8 daily consumption variables were reduced to two components: (a) Vegetables (fruit and vegetables) and (b) Carbohydrates (pasta, rice, and cereals). 

A first descriptive bivariate analysis was carried out among the variables of most interest to study the relationships between them. 

The statistical analysis was based on logistic regression, appropriate for working with a binary variable, and allowing us to model the likelihood of doing shift work or not as a function of a series of explanatory variables. In this way, we calculated the associations between a dichotomous variable (working shifts or not) and a series of covariates of interest that allowed us to adjust for the primary care workers’ sex and daily habits.

A general model considering the entire study population was made to detect the relationship between the variables analyzed and working shifts or not. Then, considering the different distribution by sex and different age group patterns observed in this first analysis, a sensitivity analysis was performed to check the results. Hence, the analyses were repeated, stratifying by sex, and then also stratifying by age group.

The Strengthening the Reporting of Observational Studies in Epidemiology (STROBE) [24] checklist was used to ensure the rigour of the research.

## 3. Results

The study response rate was 68%. Of the participating nurses, 86.3% were female and the majority were over 35 years of age (70.3%). In terms of experience in primary care, 58.4% had between 11 and 35 years of experience and 21% had between 6 and 10 years. Forty-two-point-nine percent worked in health regions located near the sea (Table 1). 

According to the descriptive analysis, it was observed that almost 95% of the nurses in the sample reported eating an adequate or healthy diet (68.2% ate pastries less than four times a week and 82.9% ate fast food less than four times a week). Regarding physical exercise, 45.2% of the primary care nurses did not engage in any type of physical activity, 10% only one day a week, 16.9% two days a week, and 27.9% three days a week or more. 

According to the descriptive bivariate analysis, by gender, male nurses did more exercise than female nurses (53.3% vs. 23.8%; *p* < 0.05). Regarding smoking, 85.8% of the nurses did not smoke. Of the nurses who smoked, the majority (46.7%) smoked between 11 and 20 cigarettes per day. By gender, male nurses smoked more than female nurses (23.3% vs. 12.6%). In addition, a trend was established between smoking habits and type of diet; the professionals who smoked being those who had a less healthy diet (45.5% of nurses who did not eat a healthy diet were smokers vs. 7.7% of nurses who smoked had a healthy diet). Fifty-seven-point-nine percent of the nurses had a normal body mass index and 32.8% were overweight (Table 2). 

The results of the bivariate analysis showed that 60.3% (*n* = 132) of the nurses in our sample did shift work, especially the younger ones (18–24 years: 80.8% vs. 55–65 years: 23.1%; *p* < 0.001). 

Generally speaking, nurses who did shift work had poorer eating habits; 81.8% of the nurses who ate an inadequate diet worked shifts. In contrast, 40.6% of the nurses who ate an adequate diet and 38.5% of the nurses who had a healthy diet did not work shifts. By food groups, a trend was established between a higher consumption of fast food (83.3% vs. 44.4%; *p* < 0.01), cold cuts (71.8% vs. 51.4%; *p* < 0.001), and pastries (76.8% vs. 47.3%, *p* < 0.001) among the group of nurses doing shift work. 

Regarding body mass index, there was a tendency for nurses working shifts to have abnormal weight values. Of the nurses who worked shifts, 64.9% were overweight and 68.8% were obese. In contrast, 45.9% of the nurses who did not do shift work had normal weight values (*p* = 0.05).

In relation to smoking, although there was a tendency for nurses who did shift work to smoke (66.7% vs. 33.3%), it was not significant. However, nurses who did not smoke consumed more fast food (86.2% vs. 45.9%; *p* < 0.001), more cold cuts (70.7% vs. 52.1%; *p* < 0.001), and more pastries (76.8% vs. 48.5%; *p* < 0.001) than nurses who worked shifts (Table 3).

Finally, in relation to exercise, 77% of the nurses who exercised three or more days a week did shift work (*p* < 0.001). 

The logistic regression results showed a positive association between doing shifts and physical activity levels, especially among the younger nurses. In particular, it was observed that nurses who did shift work tended to do more exercise; 2 days a week, compared to not doing any exercise at all (OR = 4, IC = 1.4–11.1). Moreover, nurses under 34 years of age were those who did most shift work (OR = 11.2; IC = 40.1–30.9), followed by nurses in the 35–44 age group (OR = 9.5; IC = 3.6–25.1) and finally those aged 45–54 (OR = 3.4; IC = 1.3–8.8). A clearly decreasing gradient existed between doing shift work and age, which was statistically significant assuming a 5% risk. Finally, no clear pattern is observed in terms of smoking diet and habit (Table 4). These results remain for women when the analysis was stratified by sex (Table 5). However, there were no significant results when stratified by age, probably due to the sample size (Table 6). In any case, these model results were already expected from the descriptive results observed at the sample level.

## 4. Discussion

The study results showed that the majority of primary care nurses surveyed had an adequate and healthy diet, low adherence to physical activity, low tobacco consumption, and a high level of nocturnal work activity. Previous studies have described unhealthy lifestyle behaviors among nurses, especially related to poor diets, low levels of exercise [5,6,16]. 

According to the WHO, it is essential that health workers engage in healthy behaviors, including: eating a healthy diet (high in fruits, vegetables, fish, nuts, and seeds); accumulating 150 min of moderate-intensity physical activity per week; maintaining low stress levels; achieving approximately 8 h of rest per day; and avoiding smoking. These behaviors reduce the risk of no communicable diseases and promote well-being [16]. However, while eating-related habits were correct, a high percentage of nurses were overweight. Previous studies have also shown a high prevalence of obesity among nurses [16]. This fact could be explained by the low adherence of nurses to physical activity. Previously, different authors have related low levels of exercise with developing chronic diseases [16,25,26]. Thus, primary care nurses lack strategies to improve adherence to physical activity in keeping with the International Council of Nurses’ call encouraging these health workers to engage in exercise [27].

According to Friis [28], nurses smoke less when compared with the general female population. In this sense, the results of our study showed the same trend, since the percentage of nurses who smoked in our study was very low. 

Findings from this study showed that shift work was directly related to age, i.e., younger nurses did more shift work. These results confirm research that found an association in particular between health professionals under 50 years of age and working unconventional work schedules, including night shifts [29].

It was also found in our study that nurses who did shift work had worse eating habits, with a higher consumption of fast food, cold cuts, and pastries. Previous studies have shown that night shifts are a barrier to following a healthy diet [10]. Working shifts has a negative effect on dietary patterns [12,13]. Nurses doing shift work tend to modify schedules, types, and amounts of food. In addition, foods are poorly distributed throughout the day and are often high in calories [30,31] and fat intake is excessive [32]. According to Liu [15], this is why a greater number of shift-working nurses are overweight, especially those who have more overnight shifts [33,34]. However, other authors have stated that the influence of shift work on nurses’ weight remains unclear [35,36]. Knowing that nurses who work at night generally have a poorer diet suggests the need to provide healthier meals during this shift. According to the Spanish National Labor Institute, managers must be more sensitive towards nurses who do shift work, especially regarding their food habits. In this sense, some recommendations such as controlled meal plans and ensuring sufficient time to eat correctly during night shifts were reported [37]. In addition, work policies should promote greater availability of healthy food options at work. Different studies have currently shown some difficulties in this line [9,10].

The logistic regression model showed that shift-working nurses performed more physical activity than those who did not work shifts, and these results differ from previous studies, which have reported irregular work shifts to be a common barrier to physical activity adherence [38,39]. In addition, working during the night reduces hours of free time for exercise [40]. The difference between our results and those of other authors could be explained by the age of the shift-working nurses in our survey, since most of them were younger than 34 years old. In this regard, younger nurses tend to have fewer family responsibilities and more time for physical activity. However, almost half of the nurses in the study did not exercise regularly and this suggests the need to promote physical activity among these professionals. According to Azad [41] strategies that increase physical activity at the workplace in a fun way seem to be helpful, so managers could include some activities to encourage nurses to lead a healthy lifestyle by doing more physical exercise. 

The difference in findings may also be due to the difference between rotating shifts and primary care on-call shifts. The shift-working nurses in our sample were working day shifts during the week with one to two overnight shifts during the same week. This represents an important difference from the majority of studies that examine the health and working conditions of these health care workers. 

Finally, although we found a tendency to smoke among nurses doing shift work in our sample, it was not statistically significant. This would make sense given the results of other studies that have described a pattern of higher consumption of tobacco and other stimulants such as caffeine among nurses doing night shifts [25,42]. 

### 4.1. Implication for Practice

According to the World Health Organization, the workplace is an ideal setting to implement health promotion initiatives to reduce non-communicable disease risk factors [17]. An awareness of this situation can guide professionals and managers to develop strategies aimed at promoting and motivating actions to encourage working conditions that favor a healthier lifestyle. This study has aimed to assess the life habits of primary care nurses in relation to their personal characteristics and whether or not they do shift work. In addition, this is the first study to describe the situation of nurses in the Girona health region. This could be a first step for a more in-depth study to better understand how the working conditions of healthcare professionals may influence the lifestyles they adopt.

### 4.2. Limitations

The main limitation of this study was that it had a cross-sectional design, which only allowed for the study of the relationship between variables without the possibility of establishing causality, even if the size of the study population was appropriate. In addition, the results reflected the nurses’ behaviors at the time of the study, which has limitations when making future projections.

Another aspect is that the nursing staff’s medical details were not available. This information would have been interesting to see if there was a relationship between working conditions and chronic diseases. It has previously been demonstrated that working conditions such as shift work increase the risk of breast cancer and stroke, but this evidence is not clear for other chronic conditions.

On the other hand, the sample size was sufficient and allowed us to take a first snapshot of the situation of nurses in the Girona health area in relation to shift work.

## 5. Conclusions

The study findings showed that primary care nurses had adequate dietary habits and low tobacco consumption. However, women especially had a low adherence to physical activity and there was a high prevalence of nurses who were overweight.

Age was directly related to doing shift work; the younger nurses being the group that did the most.

Doing shift work had a negative effect on eating habits, with an increased consumption of fast food, cold cuts, and pastries, as well as on obesity and overweight rates. However, shift work served as a protective factor in terms of adherence to physical exercise among younger nurses.

## Figures and Tables

**Table 1:** Socio-demographic and employment characteristics of the sample.

	Total Study Population (*n*: 219)
Work area	*n* (%)
North	52 (23.7)
South	73 (33.3)
Near the sea	94 (42.9)
**Gender**	
Woman	189 (86.3)
**Age (years)**	
<34	65 (29.7)
35–44	58 (26.5)
45–54	44 (20.1)
55–65	52 (23.7)
**Work experience in the same care level (years)**	
<5	31 (14.2)
6–10	46 (21)
11–20	55 (25.1)
21–30	48 (21.9)
21–35	25 (11.4)
>36	14 (6.4)

Descriptive results: *n* (%). frequency (percentage).

**Table 2:** Variables related to diet, exercise, tobacco consumption, and body mass index.

	Total Study Population (*n*: 219)
Diet	*n* (%)
Inadequate	11 (5.0)
Adequate	192 (87.7)
Healthy	13 (5.9)
Missing values	3 (1.4)
**Exercise**	
Does not do exercise	99 (45.2)
Does exercise 1 day a week	22 (10)
Does exercise 2 days a week	37 (16.9)
Does exercise 3 days a week or more	61 (27.9)
**Cigarette smoking**	
Non-smoker	189 (86.3)
Smoker (<5 cigarettes/day)	6 (2.7)
Smoker (6–10 cigarettes/day)	4 (1.8)
Smoker (11–20 cigarettes/day)	14 (6.4)
Smoker (21–30 cigarettes/day)	6 (2.7)
**Body mass index**	
Underweight	7 (3.2)
Normal weight	122 (55.7)
Overweight	74 (33.7)
Obesity I	16 (7.3)

Descriptive results: *n* (%). frequency (percentage).

**Table 3:** Relationship between doing shift work and type of diet, physical exercise, tobacco consumption, and body mass index.

	Total Study Population (*n*: 219)		
	Does Shift Work	Does Not Do Shift Work	*p* Value
**Diet**	***n* (%)**	***n* (%)**	0.33
Inadequate	9 (81.8)	2 (18.2)	
Adequate	114 (59.4)	78 (40.6)	
Healthy	8 (61.5)	5 (38.5)	
Missing values	3 (1.4)		
**Exercise**			0.001
Does not do exercise	47 (47.5)	52 (52.5)	
Does exercise 1 day a week	9 (40.9)	13 (59.1)	
Does exercise 2 days a week	29 (78.4)	8 (21.6)	
Does exercise 3 days a week or more	47 (77)	14 (23)	
**Cigarette smoking**			0.26
Non-smoker	112 (52.3)	77 (40.7)	
Smoker (<5 cigarettes/day)	2 (33.3)	4 (66.7)	
Smoker (6–10 cigarettes/day)	3 (75)	1 (25)	
Smoker (11–20 cigarettes/day)	11 (78.6)	3 (21.4)	
Smoker (21–30 cigarettes/day)	4 (66.7)	2 (33.3)	
**Body mass index**			0.05
Underweight	7 (100)	0 (0.0)	
Normal weight	66 (54.1)	56 (45.9)	
Overweight	48 (64.9)	26 (35.1)	
Obesity	11 (68.8)	5 (31.2)	

Bivariate analysis between two qualitative variables (Chi-squared distribution).

**Table 4:** Logistic regression considering shift work as a dependent variable.

	Total Study Population (*n*: 219)		
	OR	95% CI	*p* Value
**Sexe**			
Women	ref	ref	
Men	1.6	0.5–4.7	0.417
**Age (years)**			
<34	11.2	4.1–30.9	0.000
35–44	9.5	3.6–25.1	0.000
45–54	3.4	1.3–8.8	0.011
55–65	ref	ref	
**Exercise/Week**			
No exercise/week	ref	ref	
1 day/week	1.6	0.5–5	0.401
2 days/week	4.0	1.4–11.1	0.009
3 or more days/week	2.8	1.2–6.4	0.015
Diet/Week *			
Saturated fats	1.1	08–1.5	0.515
Mediterranean diet	1.3	0.9–1.8	0.179
Animal products	1.2	0.9–1.7	0.211
**Smoking**			
No	ref	ref	
Yes	1.5	0.5–4.2	0.487

OR. Odds Ratio taking the first category as a reference marked as ref; CI, confidence interval. * The three weekly diet variables are dummies and the reference category is not consuming that aliment.

**Table 5:** Logistic regression considering shift work as a dependent variable stratifying by sex.

	Total Study Population (*n*: 219)					
	Women (*n* = 189)			Men (*n* = 30)		
	OR	95% CI	*p* Value	OR	95% CI	*p* Value
**Age (years)**						
<34	9.9	3.4–28.3	0.000	362.0	0.4–292159.1	0.084
35–44	10.9	3.9–30.6	0.000	25.5	0.2–4312.9	0.216
45–54	3.0	1.1–7.9	0.026	-	-	0.999
55–65	ref	ref		ref	ref	
**Exercise/Week**						
No exercise/week	ref	ref				
1 day/week	1.5	0.5–4.8	0.478	-	-	-
2 days/week	3.5	1.2–10.1	0.022	-	-	0.999
3 or more days/week	2.4	1.−5.9	0.050	7.0	0.4–124.4	0.183
Diet/Week *						
Saturated fats	1.0	0.8–1.5	0.795	1.3	0.3–5.2	0.748
Mediterranean diet	1.3	0.9–1.8	0.238	3.6	0.4–34.9	0.276
Animal products	1.4	1–2	0.077	0.9	0.3–3.3	0.934
**Smoking**						
No	ref	ref		ref	ref	
Yes	1.4	0.4–4.5	0.561	0.4	0.−10.2	0.569

OR. Odds Ratio taking the first category as a reference marked as ref; CI, confidence interval. * The three weekly diet variables are dummies and the reference category is not consuming that aliment.

**Table 6:** Logistic regression considering shift work as a dependent variable stratifying by age.

	Total Study Population (*n*: 219)											
	<34 (*n* = 65)			35–44 (*n* = 58)			45–54 (*n* = 44)			55–65 (*n* = 52)		
	OR	95% CI	*p* Value	OR	95% CI	*p* Value	OR	95% CI	*p* Value	OR	95% CI	*p* Value
**Gender**												
Woman	ref	ref		ref	ref		ref	ref		ref	ref	
Man	5.37	0.4–65.3	0.188	-	-	0.999	0.4	0.1–2.7	0.330	6.0	0.2–147	0.273
**Exercise/Week**												
No exercise/week	ref	ref		ref	ref		ref	ref		ref	ref	
1 day/week	-	0.4–69.7	1.000	0.27	0–3.1	0.288	0.3	0–2.8	0.265	6.7	0.9–50.4	0.063
2 days/week	5.26	0.4–22	0.208	3.11	0.2–49.6	0.423	-	-	0.999	0.6	0–8.2	0.677
3 or more days/week	2.85	-	0.315	6.17	0.5–69.5	0.141	2.0	0.4–11.7	0.422	0.3	0–5.7	0.452
Diet/Week *												
Saturated fats	3106.7	-	0.114	0.59	0.3–1.3	0.214	1.2	0.6–2.3	0.627	0.9	0.1–6.2	0.877
Mediterranean diet	3.41	0.8–13.9	0.088	1.49	0.7–3.3	0.337	1.1	0.4–2.7	0.849	0.6	0.2–1.4	0.217
Animal products	8.72	0.6–138.1	0.124	1.96	0.8–4.9	0.154	1.7	0.6–4.6	0.299	0.6	0.2–1.6	0.273
**Smoking**												
No	ref	ref		ref	ref		ref	ref		ref	ref	
Yes	4.39	0.2–84.4	0.326	0.71	0.1–6.7	0.767	0.2	0–1.3	0.088	3.4	0.3–41.4	0.334

OR. Odds Ratio taking the first category as a reference marked as ref; CI, confidence interval. * The three weekly diet variables are dummies and the reference category is not consuming that aliment.

## Data Availability

The data presented in this study are available on request from the corresponding author. Data are not publicly available due to the privacy term signed by the participants in the informed consent.

## Appendix A

QUESTIONNAIRE: “The health habits of primary care health professionals”

1. Sex?

○ Male

○ Female

2. How old are you?

○ 18-24 years old

○ 25-34 years old

○ 35-44 years old

○ 45-54 years old

○ 55-65 years old

3. What is your approximate height in cm?

4. What is your approximate weight in kg?

5. Which primary care health region do you work in?

○ North

○ South

○ Near the sea

6. What is your job category?

○ Nurse

○ Family doctor

○ Pediatrician

7. Do you do continuous on-call shifts?

○ Yes

○ No

8. How many years have you been working in primary care?

○ Less than 5 years

○ 6 to 10 years

○ 11 to 20 years

○ 21 to 30 years

○ 31 to 35 years

○ More than 36 years

9. Do you smoke?

○ Yes

○ No

10. How many days a week do you walk at a brisk pace?

○ 1 day

○ 2 days

○ 3 days or more

○ none (you don’t need to answer question no.11)

11. How many minutes on average per day?

○ 15 to 30 min

○ 30 to 45 min

○ More than 60 min

12. How many days a week do you take a leisurely walk?

○ 1 day

○ 2 days

○ 3 days or more

○ none (you don’t need to answer question no.13)

13. How many minutes on average per day?

○ Less than 30 min

○ 30 to 60 min

○ More than 60 min

14. How many days a week do you exercise?

○ 1 day

○ 2 days

○ 3 days or more

○ none (you don’t need to answer question no.15)

15. How many minutes on average per day?

○ Less than 30 min

○ 30 to 60 min

○ More than 60 min

16. Put a cross in the box that most closely matches your current eating habits.

**Table A1:** Eating habits (a).

	Less Than Once a Day	Once a Day	Twice a Day
Bread			
Vegetables/salad			
Fruit			
Milk/yoghurt			
Pasta/rice			
Olive oil			
Cereals			
1 alcoholic drink			

**Table A2:** Eating habits (b).

	Less Than 4 Times/Week	4 to 6 Times/Week	7 Times or More per Week
1 piece of meat			
Cheese			
Cold cuts			
Pastries			
Butter			
Vegetable oils			
Fast food			

**Table A3:** Eating habits (c).

	Less Than 2 Times/Week	2 or 3 Times/Week	4 Times or More per Week
1 piece of fish			
Pulses			
Nuts and dried fruit			
